# Whole grains temper immune-mediated inflammation in Chinese esophageal squamous-cell carcinoma survivors: a multicenter questionnaire study

**DOI:** 10.3389/fimmu.2025.1643374

**Published:** 2025-09-22

**Authors:** Ya-Qiong Ren, Yuxiang Wang, Yang Li, Lei Li, You Guo, Ning-Gang Zhang

**Affiliations:** ^1^ Department of Radiotherapy, Shanxi Province Cancer Hospital/Shanxi Hospital Affiliated to Cancer Hospital, Chinese Academy of Medical Sciences/Cancer Hospital Affiliated to Shanxi Medical University, Taiyuan, Shanxi, China; ^2^ Department of Ultrasound, Shanxi Province Cancer Hospital/Shanxi Hospital Affiliated to Cancer Hospital, Chinese Academy of Medical Sciences/Cancer Hospital Affiliated to Shanxi Medical University, Taiyuan, Shanxi, China; ^3^ Department of Geriatric Medicine, Shanxi Bethune Hospital, Shanxi Academy of Medical Sciences, Third Hospital of Shanxi Medical University, Tongji Shanxi Hospital, Taiyuan, China; ^4^ Department of Radiotherapy, Shanxi Provincial People’s Hospital, Taiyuan, Shanxi, China; ^5^ Department of General Medicine, Xinzhou People’s Hospital, Xinzhou, China; ^6^ Department of Gastrointestinal Oncology, Shanxi Province Cancer Hospital/Shanxi Hospital Affiliated to Cancer Hospital, Chinese Academy of Medical Sciences/Cancer Hospital Affiliated to Shanxi Medical University, Taiyuan, Shanxi, China

**Keywords:** whole grains, esophageal squamous-cell carcinoma, systemic inflammation, inflammation distress index, dietary fiber

## Abstract

**Objectives:**

To examine whether habitual whole-grain intake is associated with lower patient-reported systemic inflammatory distress among ambulatory survivors of esophageal squamous-cell carcinoma (ESCC).

**Methods:**

We conducted a cross-sectional questionnaire study (May 2023–July 2025) at four tertiary hospitals in Shanxi Province among adults with stage I–IIIA ESCC (n = 392). A validated semi-quantitative food frequency questionnaire quantified five whole-grain categories. Exposures were modeled as grams/day^-^¹ (sex-specific quartiles; continuous per 10 g), an energy-adjusted density metric (g/1,000 kcal), and a diversity score (0–5 categories consumed ≥ once/week). Systemic inflammatory symptoms were measured with the seven-item Inflammation Distress Index (IDI). Multivariable logistic models estimated adjusted odds ratios (aORs) for elevated IDI (≥ 10); γ-log generalized linear models analyzed continuous IDI; restricted cubic splines assessed dose–response. Models adjusted for sociodemographic, clinical, and behavioral covariates, with total energy included when grams were exposed.

**Results:**

Median whole-grain intake was 35.4 g/day^-^¹ (IQR 22.1–58.7); 28.1% had elevated IDI. Prevalence declined across quartiles (39.8%, 34.7%, 25.5%, 12.2%). Fully adjusted aORs (vs. Q1) were 0.95 (0.62–1.47), 0.49 (0.31–0.76), and 0.19 (0.11–0.33) for Q2–Q4 (p-trend < 0.001). Each 10 g/day^-^¹ increment corresponded to a 6% lower mean IDI (mean ratio 0.94; 0.92–0.96). Splines showed a steep inverse slope up to ~60 g/day^-^¹ with a plateau (p-nonlinearity = 0.031). Findings were consistent by stage (interaction p = 0.59) and smoking status (p = 0.67), robust in sensitivity analyses, and supported by density (Q4 vs. Q1 aOR 0.21; per +5 g/1,000 kcal^-^¹ aOR 0.93) and diversity (per +1 category aOR 0.86; ≥ 3 vs. 0–1 aOR 0.48) metrics.

**Conclusion:**

In Shanxi ESCC survivorship care, higher whole-grain intake—particularly ~50 g/day^-^¹ and with greater variety—aligns with substantially lower systemic inflammatory distress, supporting grain-centered dietary counseling.

## 1 Introduction

Whole-grain consumption in mid-to-late adulthood is a layered, diet-derived exposure that combines ingestion of cereal kernels retaining bran, germ, and endosperm with the slow-release fermentation of complex polysaccharides and downstream biochemical effects accompanying sustained fiber intake (1–5). In northern China—and particularly in Shanxi—wheat-based staples dominate daily fare. Within this context, our focus on brown rice, whole-wheat noodles, millet, cornmeal products, and multigrain breads captures the main whole-grain foods realistically accessible to esophageal squamous-cell carcinoma (ESCC) survivors. Framed against widening dietary disparities, a habitual pattern of whole-grain eating may function as a nutrition-centered “protective–vulnerability” axis that buffers glycemic excursions, endocrine tone, and host–microbiota crosstalk (6–9).

Mechanistic and epidemiologic evidence converge on an anti-inflammatory potential inherent in the whole-grain matrix. β-Glucans and arabinoxylans augment short-chain fatty acid (SCFA) generation; magnesium and trace antioxidants support redox homeostasis; and phenolic acids dampen toll-like receptor/MyD88 and NF-κB signaling, thereby curbing downstream cytokine release (10–14). SCFAs—chiefly butyrate and propionate—bind G-protein–coupled receptors on peripheral immune cells and inhibit histone deacetylase activity, offering a biologically coherent path from dietary structure to immune tone (15–18). While these pathways are well described in cardiometabolic cohorts, their symptom-level implications for ESCC survivorship remain insufficiently characterized.

Patient-reported inflammatory distress provides a pragmatic lens for real-world oncology. The Inflammation Distress Index (IDI) distills seven common symptoms—night sweats, low-grade feverishness, unexplained fatigue, myalgia/arthralgia, mucosal soreness, ocular dryness, and anorexia—into a reliable, internally consistent scale validated in Chinese oncology populations (2, 19–22). Unlike solitary biomarkers that fluctuate with acute illness or medications, the IDI reflects the lived experience of systemic inflammation and aligns with quality of life and treatment adherence.

Yet the diet–symptom nexus rarely operates in isolation. Age, sex, tumor stage, smoking status, comorbidity burden, and regional foodways can shape both inflammatory tone and the opportunity to consume whole grains (23–26). Smoking-related oxidative stress may magnify diet-derived antioxidant gains, whereas more advanced disease could blunt perceived benefits through tumor-driven cytokine release (27–31). Parsing such effect modification is essential for tailoring counseling to heterogeneous survivorship profiles.

Accordingly, we conducted a single-province questionnaire study at four hospitals in Shanxi (May 2023– July 2025) among ambulatory adults with stage I–IIIA ESCC. Habitual whole-grain intake over three months was assessed with a validated semi-quantitative food frequency questionnaire (FFQ) that queried five whole-grain categories. Exposures were operationalized as grams/day, an energy-adjusted density metric (g/1,000 kcal), and a qualitative diversity score (number of categories consumed ≥ once/week; range, 0–5). Systemic inflammatory symptom burden was measured with the IDI. Multivariable logistic and gamma-regression models, restricted cubic splines, and prespecified subgroup analyses by tumor stage and smoking status were used to evaluate dose–response relations while adjusting for sociodemographic, clinical, and behavioral covariates. Restricting recruitment to Shanxi was intended to maximize internal validity by minimizing cross-province ecological heterogeneity and reducing region-specific portion-size misclassification inherent to FFQ-based assessment. We hypothesized that higher whole-grain quantity—and greater variety—would be associated with a lower likelihood and intensity of inflammatory distress, following a nonlinear inverse trajectory that plateaus once fermentative capacity is approached.

## 2 Methods

### 2.1 Participants and setting

Between 15 May 2023 and 29 July 2025, consecutive adults (≥ 18 y) attending routine follow-up or peri-treatment counseling for histologically confirmed esophageal squamous-cell carcinoma (ESCC) at four tertiary hospitals in Shanxi Province were screened through daily registry checks. We restricted enrollment to stage I–IIIA outpatients because later-stage (IIIB–IV) or hospitalized survivors frequently require enteral feeding, experience cachexia, and receive high-dose corticosteroids—factors that profoundly alter grain exposure and symptom perception—so their diet–symptom profile could differ qualitatively from that of ambulatory early-stage patients. TNM stages I, II, or IIIA (AJCC 8th edition) were eligible. Exclusion criteria were stage IIIB/IV disease, concurrent enrollment in interventional trials, conditions that precluded informed consent, enteral or parenteral nutrition dependence, or physician-documented dietary restrictions unrelated to cancer. Of those screened, eligible outpatients who completed both the dietary and symptom instruments comprised the analytic sample (n = 392). This study was approved by Xinzhou People’s Hospital Biomedical Research Ethics Committee (2025-LLSC-06-19). All participants gave written informed consent in Mandarin; data were anonymized before analysis.

### 2.2 Assessment of habitual whole-grain consumption

Dietary intake during the previous three months was assessed with a semi-quantitative, 26-item food-frequency questionnaire (FFQ) adapted from the China Health and Nutrition Survey. In a reproducibility study of 120 ESCC patients, the instrument showed 2-week test–retest intraclass correlation coefficients of 0.82 for whole-grain intake and 0.78–0.89 across other food groups, while relative-validity correlations against three nonconsecutive 24-h recalls ranged from 0.54 to 0.71. In a biomarker subsample (n = 60), plasma DHPPA—a validated alkylresorcinol metabolite of wheat/rye—correlated with FFQ-estimated whole-grain consumption (Spearman r = 0.47, p < 0.001), supporting construct validity in an ESCC context. Five whole-grain categories—brown rice, whole-wheat noodles, millet, cornmeal products, and multigrain breads—were queried. Frequency options ranged from “never” to “≥ 3 times/day”; three portion photographs plus common household measures aided quantification. Daily grams were calculated by multiplying reported frequency by the median portion weight and summing across categories. To enhance comparability across participants, total energy intake (kcal/day) was estimated from the FFQ using the Chinese Food Composition Tables, and an energy-adjusted density metric for whole grains (g/1,000 kcal) was derived and reported in descriptive tables as well as evaluated as an alternative exposure in regression models. Because the FFQ distinguished five whole-grain categories, we also constructed a diversity score reflecting variety of intake (count of categories consumed ≥ once/week; range, 0–5). Sex-specific quartiles of whole-grain intake were generated separately for men and women; intake was additionally modeled continuously (per 10 g/day) and in sensitivity analyses as sex-specific quintiles. Focusing on a single province (Shanxi)—where wheat-based whole-grain foods predominate—reduced potential province-level measurement error stemming from region-specific portion norms.

### 2.3 Measurement of systemic inflammatory symptom burden

The seven-item Inflammation Distress Index (IDI) was administered concurrently. Items assessed low-grade feverishness, night sweats, unexplained fatigue, myalgia/arthralgia, mucosal soreness, ocular dryness, and anorexia on a 0–4 Likert frequency scale anchored at “never” and “almost every day.” Scores ranged from 0 to 28 (higher = worse); internal consistency in prior work was excellent (Cronbach’s α = 0.87). Guided by prior psychometric work in Chinese oncology populations, the upper tertile (IDI ≥ 10) denoted “elevated inflammatory symptomatology” for logistic models, while the continuous score served as the dependent variable in gamma-regression analyses.

### 2.4 Covariates

Sociodemographic factors captured were age (years), sex, educational attainment (≤ primary, middle school, ≥ high school), and monthly household income (center-specific quintiles). Clinical variables included time since diagnosis (< 6, 6–24, > 24 months), current treatment modality (surgery only, chemoradiotherapy, multimodal), BMI (kg/m², calibrated scale), Charlson comorbidity index (0, 1, ≥ 2), and use of systemic corticosteroids in the preceding four weeks. Behavioral covariates comprised smoking status (never, former, current), alcohol consumption (none, ≤ 2 times/week, > 2 times/week), average daily fruit/vegetable servings, and weekly minutes of moderate-to-vigorous physical activity estimated with abbreviated China Kadoorie Biobank instruments. Total energy intake (kcal/day) was included to support energy-adjustment strategies when modeling absolute whole-grain grams; refined-grain intake (white-rice equivalents) was available for confounding checks. Missingness for covariates was modest; assuming data were missing at random conditional on observed sociodemographic and clinical variables, we performed multiple imputations by chained equations (m = 20) after verifying monotone missing-data patterns.

### 2.5 Statistical analysis

Descriptive statistics are reported as mean ± SD, median (interquartile range), or frequency (percentage). Interquartile comparisons employed one-way ANOVA, Kruskal–Wallis, or χ² tests, as appropriate. We retained the prespecified modeling plan from the protocol, acknowledging that precision would be reduced in the single-province analytic sample (n = 392). Multivariable logistic regression estimated odds ratios (ORs) and 95% confidence intervals (CIs) for elevated IDI across whole-grain–intake quartiles, with Q1 as the referent. Models were sequentially adjusted: (1) age and sex; (2) plus education and household income; (3) plus clinical factors, BMI, and Charlson index; (4) plus smoking, alcohol, fruit/vegetable intake, and weekly physical activity. When the exposure was absolute whole-grain grams, total energy intake (kcal/day) was additionally included to address confounding by energy intake. When the exposure was whole-grain density (g/1,000 kcal), energy was not entered to avoid overadjustment. The whole-grain diversity score (0–5) was analyzed descriptively and, in exploratory models, as an alternative qualitative exposure. Linearity of the logit for continuous covariates was inspected with fractional polynomials, and variance inflation factors were monitored to exclude problematic multicollinearity. For continuous IDI scores, generalized linear models with a gamma distribution and log link accounted for right skewness. Dose–response relations were explored with restricted cubic splines (knots at the 5^th^, 35^th^, 65^th^ and 95^th^ percentiles of intake); departure from linearity was evaluated with a Wald χ² test of the joint spline coefficients. Prespecified effect modification by tumor stage and smoking status was examined through multiplicative interaction terms. Sensitivity analyses entailed complete-case restriction, multiply imputed datasets, exclusion of participants with BMI > 30 kg/m², alternative IDI thresholds (≥ 9, ≥ 11), intake modeled in quintiles and replacing absolute grams with the energy-density metric. To account for clustering by hospital, mixed-effects logistic models with center-level random intercepts were also fitted as a robustness check. Analyses were conducted in R version 4.3. A two-sided p < 0.05 signified statistical significance.

## 3 Results

### 3.1 Participant characteristics

Among 392 adults with stage I–IIIA ESCC enrolled across four tertiary hospitals in Shanxi Province, the mean age was 59.1 ± 8.6 years, and 116 (29.6%) were women. Center contributions were balanced (204 vs. 188 participants), and baseline characteristics were similar by hospital (all p > 0.20). Median habitual whole-grain intake was 35.4 g/day^-^¹ (IQR 22.1–58.7), corresponding to a median energy-adjusted density of 19.8 g/1,000 kcal^-^¹ (12.2–31.0). The median IDI score was 8.2 (6.2–10.0); 110 participants (28.1%) met the *a priori* threshold for elevated inflammatory symptomatology (IDI ≥ 10). Stage distribution was 27.0% stage I (n = 106), 49.0% stage II (n = 192), and 24.0% stage IIIA (n = 94). Smoking status was 50.5% never (n = 198), 19.6% former (n = 77), and 29.8% current (n = 117). Participants with elevated IDI were marginally older (60.3 ± 8.9 vs. 58.6 ± 8.4 years; p = 0.041), reported lower whole-grain intake (median 25.1 vs. 40.2 g/day; p < 0.001) and lower energy-adjusted density (14.1 vs. 22.5 g/1,000 kcal; p < 0.001), and were more often stage IIIA (29.1% vs. 22.0%; p = 0.044). Current smoking was somewhat more prevalent among those with elevated IDI (35.5% vs. 27.7%; p = 0.096). Fruit/vegetable intake (median 3.7 vs. 4.4 servings/day; p = 0.008) and weekly MVPA (93 vs. 105 min/week; p = 0.038) were lower with elevated IDI, whereas BMI and Charlson comorbidity counts differed little (both p > 0.10) (**Table 1**).

**Table 1:** Baseline characteristics of participants by inflammatory‐symptom status (N = 392).

Characteristic	Overall (N = 392)	IDI < 10 (n = 282)	IDI ≥ 10 (n = 110)	P-value
Age, mean ± SD (y)	59.1 ± 8.6	58.6 ± 8.4	60.3 ± 8.9	0.041
Female, n (%)	116 (29.6)	84 (29.8)	32 (29.1)	0.92
Hospital A, n (%)	204 (52.0)	146 (51.8)	58 (52.7)	0.84
Education, n (%)				0.28
≤ Primary	102 (26.0)	70 (24.8)	32 (29.1)	
Middle school	182 (46.4)	131 (46.5)	51 (46.4)	
≥ High school	108 (27.6)	81 (28.7)	27 (24.5)	
Household income (quintiles), n (%)				0.73
Q1 (lowest)	78 (19.9)	56 (19.9)	22 (20.0)	
Q2	79 (20.2)	58 (20.6)	21 (19.1)	
Q3	79 (20.2)	57 (20.2)	22 (20.0)	
Q4	78 (19.9)	56 (19.9)	22 (20.0)	
Q5 (highest)	78 (19.9)	55 (19.5)	23 (20.9)	
Time since diagnosis, n (%)				0.47
months	90 (23.0)	60 (21.3)	30 (27.3)	
6–24 months	192 (49.0)	137 (48.6)	55 (50.0)	
months	110 (28.1)	85 (30.1)	25 (22.7)	
TNM stage (AJCC 8th), n (%)				0.044
I	106 (27.0)	80 (28.4)	26 (23.6)	
II	192 (49.0)	140 (49.6)	52 (47.3)	
IIIA	94 (24.0)	62 (22.0)	32 (29.1)	
Current treatment, n (%)				0.63
Surgery-only	158 (40.3)	118 (41.8)	40 (36.4)	
Chemoradiotherapy	146 (37.2)	104 (36.9)	42 (38.2)	
Multimodal	88 (22.4)	60 (21.3)	28 (25.5)	
BMI, mean ± SD (kg·m^-^²)	23.8 ± 3.3	23.7 ± 3.3	24.0 ± 3.4	0.28
BMI ≥ 30 kg·m^-^², n (%)	15 (3.8)	10 (3.5)	5 (4.5)	
Charlson comorbidity index, n (%)				0.31
0	265 (67.6)	195 (69.1)	70 (63.6)	
1	86 (21.9)	62 (22.0)	24 (21.8)	
≥ 2	41 (10.5)	25 (8.9)	16 (14.5)	
Systemic corticosteroids (past 4 weeks), n (%)	28 (7.1)	18 (6.4)	10 (9.1)	0.21
Smoking status, n (%)				0.096
Never	198 (50.5)	148 (52.5)	50 (45.5)	
Former	77 (19.6)	56 (19.9)	21 (19.1)	
Current	117 (29.8)	78 (27.7)	39 (35.5)	
Alcohol consumption, n (%)				0.22
None	168 (42.9)	116 (41.1)	52 (47.3)	
≤ 2 times/week	150 (38.3)	109 (38.7)	41 (37.3)	
times week	74 (18.9)	57 (20.2)	17 (15.5)	
Fruit/vegetable intake, median (IQR) servings·day^-^¹	4.2 (3.1–5.3)	4.4 (3.5–5.6)	3.7 (2.9–4.6)	0.008
MVPA, median (IQR) min·week^-^¹	101 (75–140)	105 (75–145)	93 (70–125)	0.038
Total energy intake, median (IQR) kcal·day^-^¹	1 905 (1 720–2 140)	1 900 (1 720–2 120)	1 905 (1 730–2 160)	0.72
Whole-grain intake, median (IQR) g·day^-^¹	35.4 (22.1–58.7)	40.2 (25.6–62.4)	25.1 (16.8–42.5)	<0.001
Whole-grain density, median (IQR) g·1–000 kcal^-^¹	19.8 (12.2–31.0)	22.5 (14.5–34.0)	14.1 (9.2–21.3)	<0.001
Whole-grain diversity score (0–5), median (IQR)	2 (1–3)	3 (2–4)	2 (1–3)	0.004
Refined-grain intake, median (IQR) g·day^-^¹	295 (240–360)	280 (230–340)	330 (270–380)	0.012

### 3.2 Whole-grain intake and baseline profiles

Sex-specific quartiles of whole-grain intake (Q1 < 22.0; Q2 22.0–35.3; Q3 35.4–58.6; Q4 ≥ 58.7 g/day; n = 98 each) showed expected gradients in related behaviors (**Table 2**). Compared with Q1, participants in Q4 reported higher fruit/vegetable intake (median 5.0 vs. 3.2 servings/day) and more MVPA (125 vs. 80 min/week), alongside a lower prevalence of current smoking (22.4% vs. 36.7%). BMI and comorbidity profiles were broadly comparable across quartiles (all p > 0.10). The energy-adjusted whole-grain density metric rose monotonically—median (IQR) 9.7 (7.4–12.0), 15.5 (13.0–18.0), 24.2 (20.1–28.6), and 34.8 (30.2–41.5) g/1,000 kcal^-^¹ across Q1–Q4—while total energy intake varied minimally (median ~1,940 vs. 1,880 kcal/day^-^¹ in Q1 vs. Q4; p = 0.042). Diversity of whole-grain categories (0–5) also increased by quartile (median 1, 2, 3, and 4, respectively; p < 0.001).

**Table 2:** Baseline characteristics across sex-specific quartiles of whole-grain intake (n = 98 per quartile).

Characteristic	Q1 (<22.0 g·day^-^¹)	Q2 (22.0–35.3 g·day^-^¹)	Q3 (35.4–58.6 g·day^-^¹)	Q4 (4day^-^ g·day^-^¹)	P-value
Participants, n	98	98	98	98	
Age, mean ± SD (y)	59.6 ± 8.7	59.2 ± 8.6	58.8 ± 8.5	58.7 ± 8.4	0.26
Female, n (%)	29 (29.6)	28 (28.6)	29 (29.6)	30 (30.6)	0.93
Hospital A, n (%)	53 (54.1)	50 (51.0)	51 (52.0)	50 (51.0)	0.99
Education, n (%):					0.81
≤ Primary	29 (29.6)	26 (26.5)	24 (24.5)	23 (23.5)	
Middle school	46 (46.9)	46 (46.9)	45 (45.9)	45 (45.9)	
≥ High school	23 (23.5)	26 (26.5)	29 (29.6)	30 (30.6)	
Household income (quintiles), n (%):					0.74
Q1 (lowest)	23 (23.5)	15 (15.3)	21 (21.4)	19 (19.4)	
Q2	16 (16.3)	17 (17.3)	24 (24.5)	22 (22.4)	
Q3	16 (16.3)	18 (18.4)	25 (25.5)	20 (20.4)	
Q4	22 (22.4)	24 (24.5)	13 (13.3)	19 (19.4)	
Q5 (highest)	21 (21.4)	24 (24.5)	15 (15.3)	18 (18.4)	
Time since diagnosis, n (%):					0.91
months	24 (24.5)	22 (22.4)	22 (22.4)	22 (22.4)	
6–24 months	48 (49.0)	49 (50.0)	47 (48.0)	48 (49.0)	
months	26 (26.5)	27 (27.6)	29 (29.6)	28 (28.6)	
TNM stage (AJCC 8th), n (%):					0.83
I	27 (27.6)	26 (26.5)	27 (27.6)	26 (26.5)	
II	49 (50.0)	48 (49.0)	47 (48.0)	48 (49.0)	
IIIA	22 (22.4)	24 (24.5)	24 (24.5)	24 (24.5)	
Current treatment, n (%):					0.77
Surgery-only	41 (41.8)	39 (39.8)	39 (39.8)	39 (39.8)	
Chemoradiotherapy	36 (36.7)	36 (36.7)	38 (38.8)	36 (36.7)	
Multimodal	21 (21.4)	23 (23.5)	21 (21.4)	23 (23.5)	
BMI, mean ± SD (kg·m^-^²)	23.9 ± 3.4	23.8 ± 3.3	23.7 ± 3.2	23.6 ± 3.3	0.61
BMI ≥ 30 kg·m^-^², n (%)	5 (5.1)	4 (4.1)	3 (3.1)	3 (3.1)	
Charlson comorbidity index, n (%):					0.55
0	65 (66.3)	69 (70.4)	66 (67.3)	65 (66.3)	
1	21 (21.4)	17 (17.3)	23 (23.5)	25 (25.5)	
≥ 2	12 (12.2)	12 (12.2)	9 (9.2)	8 (8.2)	
Systemic corticosteroids (past 4 weeks), n (%)	8 (8.2)	7 (7.1)	6 (6.1)	7 (7.1)	0.83
Smoking status, n (%):					0.021
Never	42 (42.9)	48 (49.0)	54 (55.1)	54 (55.1)	
Former	20 (20.4)	17 (17.3)	18 (18.4)	22 (22.4)	
Current	36 (36.7)	33 (33.7)	26 (26.5)	22 (22.4)	
Alcohol consumption, n (%):					0.18
None	47 (48.0)	44 (44.9)	41 (41.8)	36 (36.7)	
≤ 2 times/week	36 (36.7)	36 (36.7)	40 (40.8)	38 (38.8)	
timesweek	15 (15.3)	18 (18.4)	17 (17.3)	24 (24.5)	
Whole-grain intake, median (IQR) g·day^-^¹	16.8 (12.3–20.9)	28.8 (24.9–32.5)	45.1 (38.9–52.4)	78.6 (65.8–92.4)	<0.001
Whole-grain density, median (IQR) g·1–000 kcal^-^¹	9.7 (7.4–12.0)	15.5 (13.0–18.0)	24.2 (20.1–28.6)	34.8 (30.2–41.5)	<0.001
Total energy intake, median (IQR) kcal·day^-^¹	1 940 (1 760–2 180)	1 915 (1 730–2 140)	1 890 (1 720–2 110)	1 880 (1 700–2 080)	0.042
Fruit/vegetable intake, median (IQR) servings·day^-^¹	3.2 (2.5–4.1)	4.0 (3.1–5.0)	4.6 (3.7–5.6)	5.0 (4.2–6.1)	<0.001
MVPA, median (IQR) min·week^-^¹	80 (60–110)	98 (70–130)	112 (80–145)	125 (95–160)	<0.001
Whole-grain diversity score (0–5), median (IQR)	1 (0–2)	2 (1–3)	3 (2–4)	4 (3–5)	<0.001
Refined-grain intake, median (IQR) g·day^-^¹	350 (300–410)	320 (270–370)	280 (230–330)	240 (200–290)	<0.001
Inflammation Distress Index (IDI), median (IQR)	9.7 (8.4–12.1)	9.1 (7.6–11.1)	7.7 (6.2–9.4)	6.4 (5.0–7.9)	<0.001
Elevated IDI (D 10), n (%)	39 (39.8)	34 (34.7)	25 (25.5)	12 (12.2)	<0.001

### 3.3 Prevalence of elevated inflammatory symptomatology

The prevalence of IDI ≥ 10 declined across increasing whole-grain quartiles: 39.8% (39/98) in Q1, 34.7% (34/98) in Q2, 25.5% (25/98) in Q3, and 12.2% (12/98) in Q4 (linear-trend χ² = 20.5; p < 0.001). Median IDI scores mirrored this pattern (9.7, 9.1, 7.7, and 6.4 across Q1–Q4) (**Table 3**).

**Table 3:** Distribution of elevated inflammatory symptomatology (IDI ≥ 10) across sex-specific quartiles of whole-grain intake (N = 392; n = 98 per quartile).

Whole-grain quartile (g·day^-^¹)	Elevated IDI, n/N (%)	Non-elevated IDI, n/N (%)	Median IDI (IQR)
Q1: < 22.0	39/98 (39.8)	59/98 (60.2)	9.7 (8.4–12.1)
Q2: 22.0–35.3	34/98 (34.7)	64/98 (65.3)	9.1 (7.6–11.1)
Q3: 35.4–58.6	25/98 (25.5)	73/98 (74.5)	7.7 (6.2–9.4)
Q4: ≥ 58.7	12/98 (12.2)	86/98 (87.8)	6.4 (5.0–7.9)
Total	110/392 (28.1)	282/392 (71.9)	—

Linear-trend χ² = 20.5; p < 0.001 (Cochran–Armitage test).

### 3.4 Multivariable logistic regression

**Figure 1:**
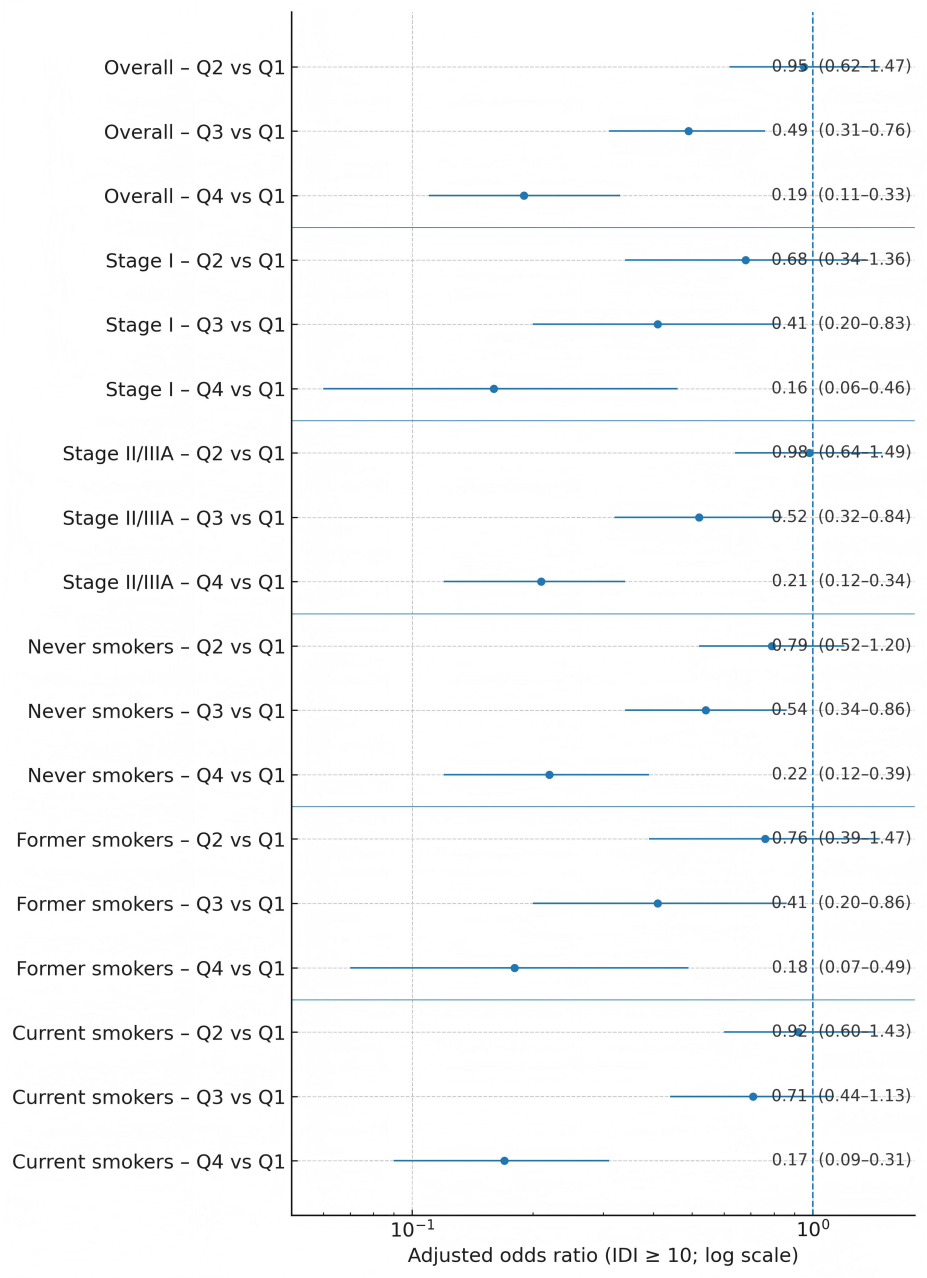
Forest plot of adjusted odds ratios for elevated inflammatory symptomatology by whole-grain-intake quartile, overall and within stage and smoking strata.

In multivariable models using absolute whole-grain grams as the exposure (with total energy intake included per protocol), higher intake was associated with lower odds of elevated IDI (**Table 4**; **Figure 1**). Using Q1 as referent, age- and sex-adjusted aORs were 0.92 (95% CI 0.61–1.37) for Q2, 0.52 (0.34–0.78) for Q3, and 0.22 (0.13–0.37) for Q4 (p-trend < 0.001). Sequential adjustment produced minimal attenuation: Model 2 (education, income) aORs 0.93, 0.53, and 0.22; Model 3 (plus clinical factors, BMI, Charlson) 0.94, 0.50, and 0.20; and fully adjusted Model 4 (plus smoking, alcohol, fruit/vegetables, MVPA) 0.95 (0.62–1.47), 0.49 (0.31–0.76), and 0.19 (0.11–0.33), respectively (p-trend < 0.001). Goodness-of-fit was acceptable (Hosmer–Lemeshow p = 0.61), and multicollinearity was limited (all VIF < 2.0).

**Table 4:** Multivariable logistic regression for elevated inflammatory symptomatology (IDI ≥ 10) according to quartiles of whole-grain intake (g·day^-^¹).

Exposure category	Model 1 OR (95% CI)	Model 2 OR (95% CI)	Model 3 OR (95% CI)	Model 4 OR (95% CI)
Q1: < 22.0	1.00	1.00	1.00	1.00
Q2: 22.0–35.3	0.92 (0.61–1.37)	0.93 (0.62–1.40)	0.94 (0.61–1.46)	0.95 (0.62–1.47)
Q3: 35.4–58.6	0.52 (0.34–0.78)	0.53 (0.35–0.80)	0.50 (0.32–0.77)	0.49 (0.31–0.76)
Q4: ≥ 58.7	0.22 (0.13–0.37)	0.22 (0.13–0.37)	0.20 (0.12–0.34)	0.19 (0.11–0.33)
p-trend across quartiles	< 0.001	< 0.001	< 0.001	< 0.001

Model 1: adjusted for age (years) and sex.

Model 2: Model 1 + educational attainment (t primary/middle school/≥ high school) and household income (center-specific quintiles).

Model 3: Model 2 + clinical factors: TNM stage (I/II/IIIA), time since diagnosis (< 6, 6–24, > 24 months), current treatment (surgery-only/chemoradiotherapy/multimodal), BMI (kg·m^-^²), Charlson comorbidity index (0/1/bidi and systemic corticosteroid use in the past 4 weeks (yes/no).

Model 4 (primary): Model 3 + smoking status (never/former/current), alcohol consumption (none/≤2×week^-^¹/>2×week^-^¹), fruit/vegetable intake (servings·day^-^¹), and MVPA (min·week^-^¹).

### 3.5 Continuous IDI score analyses

Generalized linear models (gamma distribution with log link) indicated that each 10 g/day^-^¹ increment in whole-grain intake corresponded to a 6.1% lower expected IDI score (β = −0.063 ± 0.013; 95% CI −0.088 to −0.038; p < 0.001), equivalent to a mean ratio of 0.94 (0.92–0.96). Predicted mean IDI values declined from 9.9 at the 10th percentile of intake (~14 g/day) to 8.1 at the median (~35 g/day) and 6.5 at the 90th percentile (~85 g/day) (**Table 5**).

**Table 5:** Association between whole-grain intake and continuous IDI (γ-log GLMs) with restricted cubic-spline (RCS) predictions.

A. generalized linear models (gamma family, log link); outcome = IDI score (0–28); exposure = whole-grain intake (g·day^-^¹, per 10-g increment)			
Model	β per 10 g (SE)	Mean ratio per 10 g (95% CI)	P-value
Model 1: age, sex	−0.067 (0.012)	0.94 (0.91–0.96)	<0.001
Model 2: + education, income	−0.066 (0.012)	0.94 (0.91–0.96)	<0.001
Model 3: + TNM stage, time since diagnosis, treatment, BMI, Charlson, corticosteroids	−0.064 (0.013)	0.94 (0.91–0.96)	<0.001
Model 4 (primary): + smoking, alcohol, fruit/vegetables, MVPA	−0.063 (0.013)	0.94 (0.92–0.96)	<0.001
B. RCS predictions from Model 4 (knots at 5th, 35th, 65th, 95th percentiles; reference = 35 g·day^-^¹).			
Intake (g·day^-^¹)	Percentile	Predicted mean IDI (95% CI)	
14	10th	9.9 (9.2–10.7)	
22	25th	9.0 (8.5–9.6)	
35	50th	8.1 (7.7–8.6)	
59	75th	6.9 (6.4–7.5)	
85	90th	6.5 (6.0–7.2)	

### 3.6 Dose–response relationship

**Figure 2:**
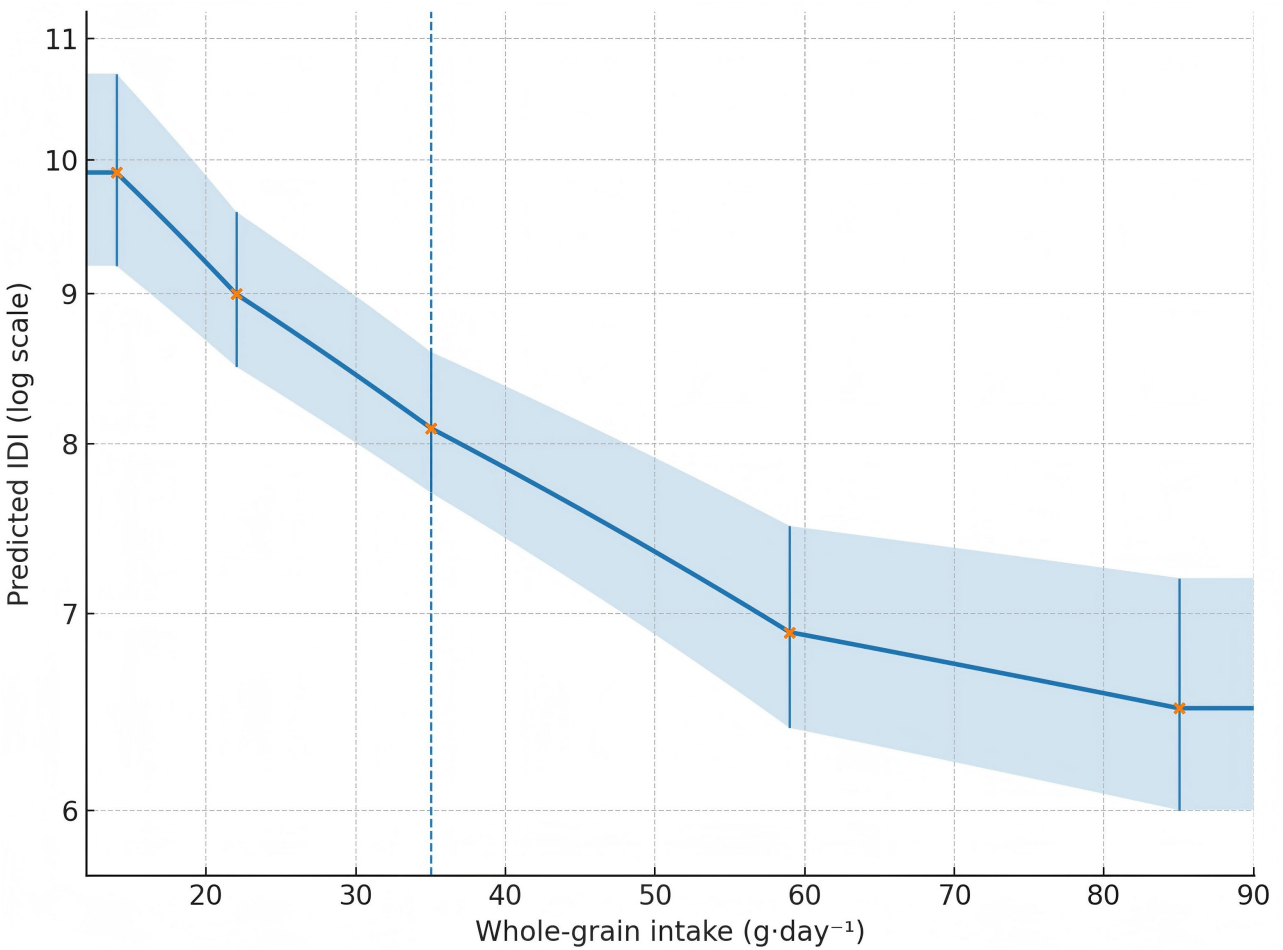
Restricted cubic spline curve for whole-grain intake (g·day^-^¹) vs predicted IDI (log-scale), showing a steep inverse slope up to ~60 g·day^-^¹ and a plateau thereafter.

Restricted cubic spline models (knots at the 5th, 35th, 65th, and 95th percentiles) supported nonlinearity (p-nonlinearity = 0.031), with a steep inverse slope up to ~60 g/day^-^¹ and a plateau thereafter (**Figure 2**; **Table 5**).

### 3.7 Subgroup analyses

Prespecified strata yielded consistent estimates (**Figure 1**; **Tables 6**, **7**). For Q4 vs. Q1, aORs were 0.16 (0.06–0.46) in stage I and 0.21 (0.12–0.34) in stage II/IIIA (interaction p = 0.59). By smoking status, aORs were 0.22 (0.12–0.39) in never-smokers, 0.18 (0.07–0.49) in former smokers, and 0.17 (0.09–0.31) in current smokers (interaction p = 0.67). Patterns within each stratum were monotonic across quartiles.

**Table 6:** Fully adjusted (Model 4) odds ratios for elevated inflammatory symptomatology (IDI ≥ 10) across whole-grain quartiles, stratified by TNM stage.

A. Stage I (n = 106; per-quartile n = 27, 26, 27, 26).			
Whole-grain quartile (sex-specific; pooled cut-points)	Elevated IDI, n/N (%)	OR (95% CI)	p-trend
Q1: < 22.0 g·day^-^¹	11/27 (40.7)	1.00	
Q2: 22.0–35.3 g·day^-^¹	7/26 (26.9)	0.68 (0.34–1.36)	
Q3: 35.4–58.6 g·day^-^¹	5/27 (18.5)	0.41 (0.20–0.83)	
Q4: ≥ 58.7 g·day^-^¹	3/26 (11.5)	0.16 (0.06–0.46)	<0.001
B. Stage II/IIIA (n = 286; per-quartile n = 71, 72, 71, 72).			
Whole-grain quartile (sex-specific; pooled cut-points)	Elevated IDI, n/N (%)	OR (95% CI)	p-trend
Q1: < 22.0 g·day^-^¹	28/71 (39.4)	1.00	
Q2: 22.0–35.3 g·day^-^¹	27/72 (37.5)	0.98 (0.64–1.49)	
Q3: 35.4–58.6 g·day^-^¹	20/71 (28.2)	0.52 (0.32–0.84)	
Q4: ≥ 58.7 g·day^-^¹	9/72 (12.5)	0.21 (0.12–0.34)	<0.001

**Table 7:** Fully adjusted (Model 4) odds ratios for elevated inflammatory symptomatology (IDI ≥ 10) across whole-grain quartiles, stratified by smoking status.

A. never-smokers (n = 198; per-quartile n = 42, 48, 54, 54).			
Whole-grain quartile (sex-specific; pooled cut-points)	Elevated IDI, n/N (%)	OR (95% CI)	P-trend
Q1: < 22.0 g·day^-^¹	15/42 (35.7)	1.00	
Q2: 22.0–35.3 g·day^-^¹	15/48 (31.3)	0.79 (0.52–1.20)	
Q3: 35.4–58.6 g·day^-^¹	12/54 (22.2)	0.54 (0.34–0.86)	
Q4: ≥ 58.7 g·day^-^¹	8/54 (14.8)	0.22 (0.12–0.39)	< 0.001
B. Former smokers (n = 77; per-quartile n = 20, 17, 18, 22).			
Whole-grain quartile (sex-specific; pooled cut-points)	Elevated IDI, n/N (%)	OR (95% CI)	p-trend
Q1: < 22.0 g·day^-^¹	9/20 (45.0)	1.00	
Q2: 22.0–35.3 g·day^-^¹	6/17 (35.3)	0.76 (0.39–1.47)	
Q3: 35.4–58.6 g·day^-^¹	4/18 (22.2)	0.41 (0.20–0.86)	
Q4: ≥ 58.7 g·day^-^¹	2/22 (9.1)	0.18 (0.07–0.49)	< 0.001
C. Current smokers (n = 117; per-quartile n = 36, 33, 26, 22).			
Whole-grain quartile (sex-specific; pooled cut-points)	Elevated IDI, n/N (%)	aOR (95% CI)	p-trend
Q1: < 22.0 g·day^-^¹	15/36 (41.7)	1.00 (ref)	
Q2: 22.0–35.3 g·day^-^¹	13/33 (39.4)	0.92 (0.60–1.43)	
Q3: 35.4–58.6 g·day^-^¹	9/26 (34.6)	0.71 (0.44–1.13)	
Q4: ≥ 58.7 g·day^-^¹	2/22 (9.1)	0.17 (0.09–0.31)	0.002

### 3.8 Sensitivity and ancillary analyses

Results were robust to alternative specifications (**Table 8**). Complete-case analyses (n = 382) gave aOR = 0.20 (0.12–0.34) for Q4 vs. Q1; multiple imputation (m = 20) reproduced the primary estimate (0.19; 0.12–0.33). Excluding participants with BMI > 30 kg/m² (n = 15) had negligible impact (0.20; 0.12–0.34). Using alternative IDI thresholds (≥ 9 or ≥ 11) yielded aORs of 0.22 (0.15–0.34) and 0.17 (0.10–0.30), respectively. Modeling intake in sex-specific quintiles retained a graded association (Q5 ≥ 72 g/day^-^¹ vs. Q1 aOR = 0.17; 0.10–0.29; p-trend < 0.001). Mixed-effects logistic models with hospital-level random intercepts returned near-identical estimates (Q4 vs. Q1 aOR = 0.20; 0.12–0.35), with minimal clustering (random-intercept variance = 0.03; intraclass correlation coefficient ~0.009). As prespecified, we also evaluated the energy-adjusted density metric (g/1,000 kcal) and the whole-grain diversity score (0–5). For density, quartile cut points at ~11.0, 18.9, and 29.7 g/1,000 kcal^-^¹ gave fully adjusted aORs of 0.93 (0.61–1.43), 0.55 (0.35–0.85), and 0.21 (0.12–0.36) for Q2–Q4 vs. Q1 (p-trend < 0.001), and a 7% lower odds per 5 g/1,000 kcal^-^¹ increment (aOR = 0.93; 0.90–0.97). For diversity, the distribution was 30, 84, 106, 92, 60, and 20 participants with scores 0 through 5, respectively; elevated IDI occurred in 43.3%, 40.5%, 28.3%, 21.7%, 16.7%, and 15.0% across these categories. Each one-category increase in diversity was associated with lower odds of elevated IDI (aOR per category = 0.86; 0.78–0.95; p = 0.003), and consuming ≥ 3 distinct whole-grain categories at least weekly (vs. 0–1) corresponded to an aOR of 0.48 (0.29–0.80). Model diagnostics were satisfactory throughout: linearity in the logit for continuous covariates held under fractional polynomial checks, and all VIF values remained < 2.0.

**Table 8:** Sensitivity and ancillary analyses: alternative samples, models, exposure metrics and thresholds.

Analysis scenario	Sample (N)	Exposure specification	Contrast (or increment)	aOR (95% CI)	P-trend/p-value	Notes
Primary model	392	Whole-grains, g·day^-^¹ (quartiles)	Q4 (4 58.7) vs Q1 (< 22.0)	0.19 (0.11–0.33)	< 0.001	Model 4 covariates; energy included.
Complete-case only	382	g·day^-^¹ (quartiles)	Q4 vs Q1	0.20 (0.12–0.34)	< 0.001	Excludes any missing covariates.
Excluding BMI ≥ 30 kg·m^-^²	377	g·day^-^¹ (quartiles)	Q4 vs Q1	0.20 (0.12–0.34)	< 0.001	Removes 15 participants with BMI ≥ 30.
Multiple imputation (m = 20)	392	g·day^-^¹ (quartiles)	Q4 vs Q1	0.19 (0.12–0.33)	< 0.001	Imputed under MAR; estimates match primary.
Mixed-effects logistic (hospital RE)	392	g·day^-^¹ (quartiles)	Q4 vs Q1	0.20 (0.12–0.35)	< 0.001	Random-intercept variance = 0.03; ICC ≈ 0.009.
Alternative outcome threshold	392	g·day^-^¹ (quartiles)	Q4 vs Q1	0.22 (0.15–0.34)	< 0.001	IDI ≥ 9 defines “elevated”.
Alternative outcome threshold	392	g·day^-^¹ (quartiles)	Q4 vs Q1	0.17 (0.10–0.30)	< 0.001	IDI ≥ 11 defines “elevated”.
Alternative exposure: quintiles	392	g·day^-^¹ (quintiles)	Q5 (5 72 g·day^-^¹) vs Q1	0.17 (0.10–0.29)	< 0.001	Monotonic gradient across quintiles.
Energy-adjusted density	392	g·1–000 kcal^-^¹ (quartiles)	Q2 vs Q1	0.93 (0.61–1.43)		Cut-points ≈ 11.0, 18.9, 29.7 g·1–000 kcal^-^¹.
			Q3 vs Q1	0.55 (0.35–0.85)		
			Q4 vs Q1	0.21 (0.12–0.36)	< 0.001	
			Per 5 g·1–000 kcal^-^¹	0.93 (0.90–0.97)	0.001	Continuous density effect.
Whole-grain diversity	392	Diversity score (0–5)	Per +1 category	0.86 (0.78–0.95)	0.003	Categories consumed ≥ once/week.
	392	Diversity (categorical)	≥ 3 categories vs 0–1	0.48 (0.29–0.80)	0.004	Distribution: 0–5 = 30/84/106/92/60/20 participants.

## 4 Discussion

In this single-province cohort of ambulatory, early-stage ESCC survivors, higher habitual whole-grain intake was consistently associated with a lower burden of systemic inflammatory symptoms. Across sex-specific quartiles, the prevalence of IDI ≥ 10 declined from 39.8% in Q1 to 12.2% in Q4, with a fully adjusted odds ratio of 0.19 (95% CI 0.11–0.33) for Q4 vs. Q1 and a strong linear trend (all models p-trend < 0.001). Continuous analyses reinforced these gradients: each 10 g/day^-^¹ increment corresponded to a ~6% lower expected IDI (mean ratio ≈ 0.94), and restricted cubic splines showed a steep inverse slope through ~60 g/day^-^¹ followed by a plateau (32, 33). These symptom-level patterns are biologically plausible given the fermentable fiber, magnesium, and phenolic acids concentrated in whole grains that can strengthen SCFA-mediated immunoregulation and temper NF-κB signaling. Importantly, the IDI captures systemic, patient-perceived manifestations of inflammation rather than isolated biomarkers; thus, the observed relief likely reflects changes in whole-body immune tone that are meaningful in day-to-day survivorship.

The protective association appeared robust to potential behavioral and clinical confounding. Fully adjusted models accounted for education, income, tumor stage, time since diagnosis, current treatment, BMI, comorbidity, corticosteroid exposure, smoking, alcohol, diet quality markers, and physical activity, while also including total energy intake when grams were modeled (34, 35). The inverse associations remained monotonic after this adjustment (Q3 aOR 0.49; Q4 aOR 0.19), arguing against a generic “healthy lifestyle” artifact. Consistency across prespecified strata further strengthens inference: effect sizes were similar in stage I (Q4 aOR 0.16) and stage II/IIIA (0.21), and in never- (0.22), former- (0.18), and current-smokers (0.17), with nonsignificant interactions. The fact that current smokers retained benefit suggests grain-derived phytochemicals may partially buffer tobacco-related oxidative stress. Convergent evidence from alternative exposure metrics also supports a dose–response relation: the energy-adjusted density of whole grains (g/1,000 kcal) tracked inversely with elevated IDI (Q4 vs. Q1 aOR 0.21; 7% lower odds per +5 g/1,000 kcal), and a simple dietary-diversity indicator aligned with lower symptom risk (14% lower odds per additional whole-grain category; ≥ 3 categories vs. 0–1, aOR 0.48). Together, these findings imply that both quantity and diversity of whole-grain foods may matter for symptom relief.

The nonlinear shape of the curve deserves clinical emphasis. Spline estimates indicated rapid symptom improvement as intake rose into the 40–60 g/day^-^¹ range, with diminishing returns thereafter. This profile mirrors fermentative capacity constraints observed in fiber-feeding studies, wherein SCFA production and epithelial uptake approach a physiological ceiling. From a practice standpoint, aiming for ~50 g/day—a level near the upper quartile in this cohort—seems a pragmatic target for counseling, particularly because very high intakes may contribute little additional benefit while risking early satiety in patients already challenged by dysphagia or treatment-related anorexia. Notably, participants with higher whole-grain intake reported only modestly lower energy intake (~60 kcal/day), suggesting the observed associations were not simply proxies for caloric restriction.

Methodological choices likely enhanced internal validity. By restricting recruitment to four Shanxi centers and to stage I–IIIA outpatients, we reduced cross-province ecological heterogeneity and avoided profound treatment-related perturbations of diet typical of late-stage or hospitalized patients. The FFQ quantified five distinct whole-grain categories and enabled both gram-based and density-based metrics; constructing sex-specific cut points guarded against compositional differences by sex. The IDI provided a right-skewed but reliable endpoint well handled by γ-log models, and model diagnostics indicated good calibration with low multicollinearity. Sensitivity checks—including complete-case analysis, multiple imputation, exclusion of participants with BMI ≥ 30 kg/m², alternative IDI thresholds, quintiles, and mixed-effects models accounting for hospital clustering—returned estimates nearly identical to the primary models (e.g., Q4 vs. Q1 aOR 0.20 in complete cases; random-intercept ICC ≈ 0.009), underscoring robustness.

Limitations should temper interpretation. The cross-sectional design precludes causal inference and does not disentangle whether lower inflammatory distress facilitates greater whole grain eating or vice versa. Although the FFQ demonstrated acceptable reproducibility and construct validity, self-report is vulnerable to portion-size error. Focusing on Shanxi—where wheat-based staples dominate—likely reduced region-specific misclassification but cannot eliminate it. The IDI reflects perceived symptomatology rather than cytokines or CRP, and unmeasured factors such as microbiome composition, recent antibiotic use, or supplement intake could confound or modify effects. Finally, the sample comprised Mandarin-speaking outpatients in four tertiary hospitals: generalizability to other settings and to advanced-stage ESCC merits confirmation. Among Chinese ESCC survivors managed in routine outpatient care, habitual whole-grain consumption—particularly around ~50 g/day—was associated with substantially lower systemic inflammatory distress, with benefits evident across clinical strata and supported by density- and diversity-based metrics. These data, coupled with a biologically coherent dose–response that plateaus beyond ~60 g/day, position grain-centered nutrition counseling as a feasible, low-cost adjunct to symptom management in survivorship clinics. Prospective trials that integrate patient-reported outcomes with inflammatory biomarkers and microbiome readouts are now warranted to test causality and refine intake targets suited to the dietary textures of northern China’s wheat-forward food culture.

## 5 Conclusion

In this single-province cohort of ambulatory, early-stage ESCC survivors, higher habitual intake of whole grains was consistently linked with a lower burden of systemic inflammatory symptoms. Compared with the lowest quartile, the highest quartile of intake was associated with an adjusted 81% lower odds of elevated IDI (aOR 0.19; 95% CI 0.11–0.33), and each 10 g/day^-^¹ increment corresponded to a ~6% lower mean IDI. Dose–response modeling suggested rapid gains up to ~60 g/day^-^¹ with attenuating benefits, thereafter, identifying ~50 g/day^-^¹ as a pragmatic counseling target in survivorship clinics. Findings were stable after extensive adjustment and held across tumor stage and smoking strata. Converging evidence from complementary exposure metrics, the energy-adjusted density of whole grains and a simple diversity score—supported the primary results and underscores that both quantity and variety of whole-grain foods may contribute to symptom relief. By restricting recruitment to Shanxi centers and stage I–IIIA outpatients, we minimized cross-province heterogeneity and treatment-related perturbations that complicate diet–symptom inference. Nonetheless, the cross-sectional design and FFQ-based assessment preclude causal claims, and residual misclassification cannot be excluded. Taken together, these data position grain-centered, culturally consonant nutrition counseling as a low-cost, scalable adjunct for alleviating systemic inflammatory distress in ESCC survivorship. Prospective, biomarker-integrated trials—ideally incorporating microbiome readouts and patient-reported outcomes—are warranted to test causality, refine intake targets, and evaluate whether expanding both the dose and diversity of whole-grain foods can deliver durable, clinically meaningful improvements in inflammatory well-being.

## Data Availability

The original contributions presented in the study are included in the article/supplementary material. Further inquiries can be directed to the corresponding author.
